# Recurrent Cesarean Scar Ectopic Pregnancy Treated with Systemic Methotrexate

**DOI:** 10.1155/2017/9536869

**Published:** 2017-11-23

**Authors:** Chima Ndubizu, Rodney A. McLaren, Sandra McCalla, Mohamad Irani

**Affiliations:** ^1^Department of Obstetrics and Gynecology, Maimonides Medical Center, Brooklyn, NY 11219, USA; ^2^Ronald O. Perelman and Claudia Cohen Center for Reproductive Medicine, Weill Cornell Medicine, New York, NY 10021, USA

## Abstract

Cesarean scar pregnancy (CSP) is a rare event; however its incidence has been rising due to the increasing rates of cesarean deliveries. The majority of cases present with signs or symptoms requiring surgery, which often results in hysterectomy. The recurrence of CSP is even rarer with only few cases which have been reported. This is a report of recurrent cesarean scar ectopic pregnancy (RCSP) that was promptly diagnosed and managed with only systemic methotrexate. This was a 30-year-old woman, with a history of two prior cesarean deliveries followed by a CSP, who presented at 5 weeks and 3 days of gestation for her first prenatal visit. Transvaginal ultrasound revealed a RCSP. Her serum beta-human chorionic gonadotropin (*β*-hCG) level was 54,295 IU/L. The first CSP, which was diagnosed at a later stage, was treated with uterine artery embolization and systemic methotrexate leading to complete resolution within 10 weeks. The current ectopic was treated with two doses of systemic methotrexate; her serum *β*-hCG reached undetectable levels within 7 weeks. Thus, patients with a history of prior CSP should be carefully monitored with transvaginal ultrasound during subsequent pregnancies to allow early diagnosis of RCSP, which could then be treated conservatively.

## 1. Introduction

Cesarean scar pregnancy (CSP) remains a rare condition despite its increased incidence over the last two decades, which has been attributed to increased cesarean section rates and frequent use of transvaginal ultrasound in the first trimester [1]. CSP represents 6% of all ectopic pregnancies in women with at least one prior cesarean delivery [2, 3]. There are several reported cases of CSP that were managed expectantly and reached successful deliveries, mostly requiring cesarean hysterectomy [4]. Late diagnosis of CSP can be associated with devastating sequelae such as uterine rupture, hemorrhage, need for hysterectomy, and maternal death. The outcome is thus dependent on the timing of diagnosis and type of intervention.

The incidence of recurrent CSP (RCSP) is unknown but it is estimated to be much rarer than CSP [5, 6]. The reported cases were managed surgically (ultrasound-guided suction and evacuation), intragestational sac methotrexate injection, or combination of systemic methotrexate with intragestational sac injection of methotrexate and potassium chloride (KCL) [5, 7, 8]. This is a report of RCSP that was successfully treated with only systemic methotrexate.

## 2. Case Report

A 30-year-old woman G5P2113 (2 term deliveries, 1 preterm delivery, 1 CSP, and 3 living children) presented for prenatal care at 5 weeks and 3 days of gestation. The patient was asymptomatic at the time. She had a history of vaginal delivery followed by two cesarean deliveries then a CSP. The first cesarean section was performed at 31 weeks of gestation owing to the occurrence of vaginal bleeding in the setting of placenta previa. The patient underwent a second cesarean section after failing trial of labor. Her previous CSP was diagnosed at 13 weeks of gestation with *β*-hCG of 144,337 IU/L. She was treated with uterine artery embolization (UAE) and a single injection of systemic methotrexate. Serum *β*-hCG was trended until reaching undetectable levels within 10 weeks.

**Figure 1:**
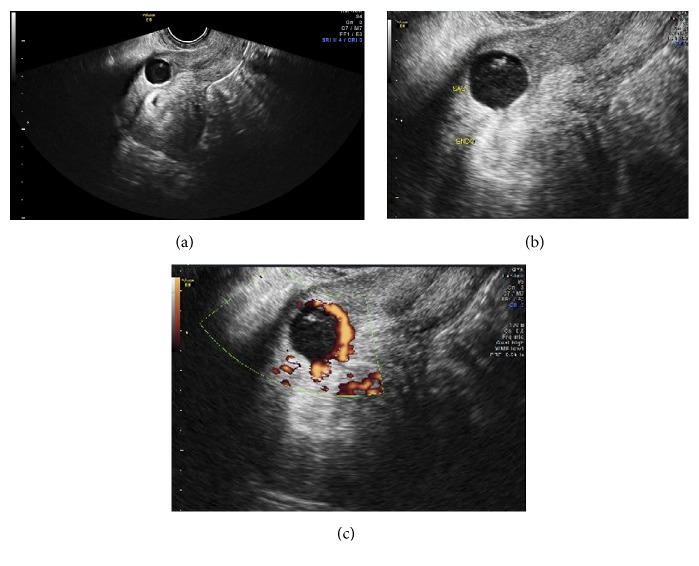
Transvaginal ultrasound showing (a) a cesarean scar ectopic pregnancy with gestational sac and yolk sac in the prior cesarean scar; (b) delineation of ectopic pregnancy and empty endometrial lining; (c) trophoblastic/placental blood flow using color Doppler.

During this visit, transvaginal ultrasound showed a CSP measuring 4 weeks and 4 days by mean sac diameter (Figure 1). A yolk sac was also visualized measuring 3.7 mm (Figure 1). On examination, patient's vital signs were within normal limits. Her abdomen was soft, nontender and not distended. There was no vaginal bleeding and the cervix was closed on speculum examination. The patient was subsequently sent to the hospital for further management.

Initial laboratory tests revealed a serum *β*-hCG of 54,295 IU/L. The hemoglobin, white blood cell count, platelet count, creatinine, and liver function enzymes were within the reference ranges. After counseling the patient about her treatment options, she elected to proceed with an intramuscular (IM) single dose regimen of methotrexate 90 mg (50 mg/m^2^). The patient was discharged home with strict precautions and advised to return to the emergency room if she develops any signs or symptoms of ruptured ectopic pregnancy. Serum *β*-hCG levels on day 4 and day 7 were 69,466 IU/L and 64,963 IU/L, respectively. Therefore, a second dose of IM methotrexate was administered. Serum *β*-hCG levels dropped appropriately from 50,042 IU/L on day 4 to 35,381 IU/L on day 7. Patient remained asymptomatic with normal vital signs. *β*-hCG was monitored weekly until becoming undetectable within 7 weeks.

## 3. Discussion 

CSP is one of the rarest forms of all ectopic pregnancies with an incidence ranging from 1/2216 to 1/800 pregnancies [1]. The exact pathophysiology of a cesarean scar ectopic is unknown; however the most common theory is the development of a scar defect or microscopic dehiscence in the scar secondary to fibrosis and poor vascularization leading to compromised wound healing [2]. Maymon et al. have suggested that repairing the uterine incision with a single noninverted running suture may impair postoperative healing and subsequently give rise to more scar defects compared to double layer closure [9]. Moreover, some investigators have proposed that higher number of cesarean deliveries might be associated with higher risk of fibrosis and abnormal healing and thus higher incidence of CSP [2]. Vervoort et al. have proposed four possible hypotheses for the development of uterine scar defects: (1) low (cervical) location of the uterine incision, (2) inadequate closure of the uterine wall, (3) surgical activities that may induce adhesion formation, and (4) patient-related factors that may hamper wound healing [10]. Our patient had two prior cesarean sections with uterine incisions that were repaired with a single layer suture, which may have increased her risk for CSP.

The clinical manifestations of CSP are similar to tubal and cervical ectopic pregnancies. Transvaginal ultrasound is extremely helpful to diagnose CSP using the following criteria: (1) an empty endometrial cavity and cervical canal; (2) a gestational sac identified in the anterior uterine wall; and (3) prominent trophoblastic/placental blood flow [11]. Given the history of prior CSP, our patient underwent an early ultrasound confirming the diagnosis of RCSP.

Pregnancies implanting in cesarean scars are divided into two types [12]. Type 1 results from the progression of the pregnancy into the uterine cavity. This particular type has led to viable births following the diagnosis of CSP [4, 12]. Type 2 results from deeper implantation of the amniotic sac into the scar with progression towards uterine rupture during the first trimester of pregnancy. Vial et al. have identified the following sonographic criteria to establish the diagnosis of type 2 CSP: (1) the trophoblast predominantly located between the bladder and the anterior uterine wall, (2) no fetal parts visualized in the uterine cavity, and (3) a sagittal view of the uterus showing a discontinuity in the anterior wall of the uterus caused by the amniotic sac [12]. Our patient had type 2 CSP and thus termination of pregnancy was recommended.

Recent evidence has demonstrated that CSP is a precursor to morbidly adherent placenta (MAP) [4, 13]. MAP is an obstetric complication where the placenta invades into the myometrium and can lead to significant maternal morbidity and mortality. It has been suggested that elective cesarean hysterectomy without removing the placenta decreases maternal morbidity [14]. Timor-Tritsch et al. reported 9 cases of CSP that were diagnosed in the first trimester and resulted in live births but needed hysterectomies due to MAP [4]. Interestingly, CSP and MAP share similar histopathological features [13]. Cali et al. recently proposed a new ultrasound sign “crossover sign” to predict the severity of MAP that develop with CSPs that are diagnosed in the first trimester [15]. In light of the current evidence, prospective trials are needed to determine whether counseling patients with CSP needs to be modified and whether expectant management can be offered along with planned cesarean hysterectomy.

The management of CSP has been suggested to correlate with the recurrence rate of CSP [8]. Ben Nagi et al. reported a case of three consecutive CSP; the first two CSP were treated by suction and evacuation while the third one was managed by a laparotomy to completely excise the deficient scar and repair the uterine wall [8]. Following this repair, the patient had two intrauterine pregnancies that implanted in normal location within the uterine cavity. The authors suggested, based on that case, that repair of uterine defect may reduce the recurrence rate of CSP [8]. However, the lack of sufficient evidence of beneficial impact and/or potential complications associated with laparotomy, such as adhesions and possibility of poor scar healing, is precluded considering repair of defect as first-line treatment for CSP in hemodynamically stable patients. Our patient was managed conservatively twice, thus avoiding the need for laparotomy.

There are no guidelines for the management of CSP or RCSP. Besides suction and evacuation, the reported conservative managements for RCSP are intragestational administration of methotrexate with or without KCL and systematic methotrexate [5, 7, 8]. Several studies have recommended against conservative management of CSP. Washburn et al. reported a 62% failure rate of single dose therapy of systemic methotrexate or intrasac KCl in 8 cases of CSP [16]. Birch Petersen et al. reported 25% of patients (*n* = 339) with CSP treated with systemic methotrexate required additional therapy [17]. In a review of 1647 of patients with CSP, 144 cases out of 559 were successfully treated with systemic methotrexate without additional therapy. Higher number of prior cesarean deliveries, higher parity, lower gestational age, and absence of bleeding or embryonic cardiac activity were significantly associated with successful response to systemic methotrexate [18]. Our patient, which exhibited all these factors, illustrates that systemic methotrexate can successfully treat RCSP even when serum *β*-hCG levels reach 50.000 IU/L. Therefore, selecting good candidates for systemic methotrexate along with providing appropriate counseling are required to attain desirable outcomes.

There are only few small studies investigating the reproductive outcomes following successful treatment of CSP [6, 19]. Maymon et al. reviewed the records of 18 women who were treated for CSP between 2000 and 2009 [19]. The authors showed that 7 of the 8 patients who attempted to conceive became pregnant naturally while 1 of them conceived by in vitro fertilization/intracytoplasmic sperm injection. Two patients (25%) had RCSP and the remaining six delivered by repeat cesarean deliveries [19]. Similarly, Ben Nagi et al. followed the outcomes of 24 women who were treated for CSP between 1999 and 2005 [6]. The findings were reassuring as the majority (88%) conceived naturally (mostly within one year of attempt), while only 1 of the 21 patients had RCSP [6]. Likewise, Washburn et al. managed 23 patients with CSP and reported 48% documented subsequent pregnancy with 82% live birth rate [16].

Patients with a history of cesarean delivery should be carefully monitored during subsequent pregnancies to allow early diagnosis of CSP. Patients who are carefully selected can be treated by varied conservative therapies. Further studies investigating the effect of different therapeutic modalities of CSP on fertility, placentation, and outcomes of future pregnancies are needed.

## References

[1] Rotas M. A., Haberman S., Levgur M. (2006). Cesarean scar ectopic pregnancies: etiology, diagnosis, and management. *Obstetrics & Gynecology*.

[2] Jurkovic D., Hillaby K., Woelfer B., Lawrence A., Salim R., Elson C. J. (2003). First-trimester diagnosis and management of pregnancies implanted into the lower uterine segment Cesarean section scar. *Ultrasound in Obstetrics & Gynecology*.

[3] Seow K.-M., Huang L.-W., Lin Y.-H., Lin M. Y.-S., Tsai Y.-L., Hwang J.-L. (2004). Cesarean scar pregnancy: issues in management. *Ultrasound in Obstetrics & Gynecology*.

[4] Timor-Tritsch I. E., Monteagudo A., Cali G. (2014). Cesarean scar pregnancy is a precursor of morbidly adherent placenta. *Ultrasound Obstet Gynecol*.

[5] Lee J. H., Kwon D. H., Ahn K. H., Hong S. C., Kim T. (2016). Concomitant ultrasound-guided intra-gestational sac methotrexate-potassium chloride and systemic methotrexate injection in the recurrent cesarean scar pregnancy. *Obstetrics & Gynecology Science*.

[6] Ben Nagi J., Helmy S., Ofili-Yebovi D., Yazbek J., Sawyer E., Jurkovic D. (2007). Reproductive outcomes of women with a previous history of Caesarean scar ectopic pregnancies. *Human Reproduction*.

[7] Hasegawa J., Ichizuka K., Matsuoka R., Otsuki K., Sekizawa A., Okai T. (2005). Limitations of conservative treatment for repeat Cesarean scar pregnancy. *Ultrasound in Obstetrics & Gynecology*.

[8] Ben Nagi J., Ofili-Yebovi D., Sawyer E., Aplin J., Jurkovic D. (2006). Successful treatment of a recurrent Cesarean scar ectopic pregnancy by surgical repair of the uterine defect. *Ultrasound in Obstetrics & Gynecology*.

[9] Maymon R., Halperin R., Mendlovic S., Schneider D., Herman A. (2004). Ectopic pregnancies in a caesarean scar: review of the medical approach to an iatrogenic complication. *Human Reproduction Update*.

[10] Vervoort A. J. M. W., Uittenbogaard L. B., Hehenkamp W. J. K., Brölmann H. A. M., Mol B. W. J., Huirne J. A. F. (2015). Why do niches develop in Caesarean uterine scars? Hypotheses on the aetiology of niche development. *Human Reproduction*.

[11] Seow K.-M., Hwang J.-L., Tsai Y.-L. (2001). Ultrasound diagnosis of a pregnancy in a Cesarean section scar. *Ultrasound in Obstetrics & Gynecology*.

[12] Vial Y., Petignat P., Hohlfeld P. (2000). Pregnancy in a cesarean scar. *Ultrasound in Obstetrics & Gynecology*.

[13] Timor-Tritsch I. E., Monteagudo A., Cali G. (2014). Cesarean scar pregnancy and early placenta accreta share common histology. *Ultrasound in Obstetrics & Gynecology*.

[14] Shamshirsaz A. A., Fox K. A., Erfani H. (2017). Multidisciplinary team learning in the management of the morbidly adherent placenta: outcome improvements over time. *American Journal of Obstetrics & Gynecology*.

[15] Cali G., Forlani F., Timor-Tritsch I. E., Palacios-Jaraquemada J., Minneci G., D'Antonio F. (2017). Natural history of Cesarean scar pregnancy on prenatal ultrasound: the crossover sign. *Ultrasound in Obstetrics & Gynecology*.

[16] Washburn E. E., Pocius K., Carusi D. (2017). Outcomes of nonsurgical versus surgical treatment of cesarean scar pregnancies in the first trimester. *Archives of Gynecology and Obstetrics*.

[17] Birch Petersen K., Hoffmann E., Rifbjerg Larsen C., Nielsen H. S. (2016). Cesarean scar pregnancy: a systematic review of treatment studies. *Fertility and Sterility*.

[18] Kanat-Pektas M., Bodur S., Dundar O., Bakır V. L. (2016). Systematic review: what is the best first-line approach for cesarean section ectopic pregnancy?. *Taiwanese Journal of Obstetrics and Gynecology*.

[19] Maymon R., Svirsky R., Smorgick N. (2011). Fertility performance and obstetric outcomes among women with previous cesarean scar pregnancy. *Journal of Ultrasound in Medicine*.

